# The Antiviral Drug Efavirenz in Breast Cancer Stem Cell Therapy

**DOI:** 10.3390/cancers13246232

**Published:** 2021-12-11

**Authors:** Pey-Tsyr Chiou, Stephen Ohms, Philip G. Board, Jane E. Dahlstrom, Danny Rangasamy, Marco G. Casarotto

**Affiliations:** 1 The John Curtin School of Medical Research, The Australian National University, Canberra, ACT 2600, Australia; pey-tsyr.chiou@anu.edu.au (P.-T.C.); Stephen.Ohms@aihw.gov.au (S.O.); Philip.Board@anu.edu.au (P.G.B.); danny.rangasamy@anu.edu.au (D.R.); 2 Anatomical Pathology, ACT Pathology, Canberra Hospital, Canberra, ACT 2600, Australia; jane.dahlstrom@anu.edu.au; 3 ANU Medical School, ANU College of Health and Medicine, The Australian National University, Canberra, ACT 2600, Australia

**Keywords:** breast cancer, cancer stem cells, Efavirenz, genes, treatment

## Abstract

**Simple Summary:**

Cancer stem cells (CSCs) are responsible for tumour initiation, chemo- and radiotherapy resistance and cancer recurrence. CSCs display plasticity that enables them to alter their phenotype and function making them challenging to eliminate. In this study we explore the effects of an antiretroviral medication used to treat HIV/AIDS (Efavirenz) on cancer stem cells derived from multiple breast cancer cell lines. Efavirenz has been previously found to be effective in the treatment of triple-negative breast cancers, and here we show that it is also capable of altering CSC numbers, cell morphology, RNA/microRNA gene expression and levels of epithelial/mesenchymal CSC subtypes. This study shows that, with Efavirenz, it is possible to not only eliminate primary breast cancer cells, but also to promote changes in cell morphology.

**Abstract:**

Although many breast cancer therapies show initial success in the treatment of the primary tumour, they often fail to eliminate a sub-population of cells known as cancer stem cells (CSCs). These cells are recognised for their self-renewal properties and for their capacity for differentiation often leading to chemo/radio-resistance. The antiviral drug Efavirenz has been shown to be effective in eliminating triple-negative breast cancer cells, and here we examine its effect on breast CSCs. The effects of Efavirenz on CSCs for several breast cancer cell lines were investigated by examining cellular changes upon drug treatment, including CSC numbers, morphology, RNA/microRNA expression and levels of epithelial/mesenchymal CSC subtypes. Efavirenz treatment resulted in a decrease in the size and number of tumorspheres and a reduction in epithelial-type CSC levels, but an increase in mesenchymal-type CSCs. Efavirenz caused upregulation of several CSC-related genes as well as *miR-21*, a CSC marker and *miR-182*, a CSC suppressor gene. We conclude that Efavirenz alters the phenotype and expression of key genes in breast CSCs, which has important potential therapeutic implications.

## 1. Introduction

Since the first report of cancer stem cells (CSCs) in leukaemia two decades ago, a vigorous debate has emerged as to the roles that CSCs play in cancer development and how this knowledge can be harnessed to treat and eliminate various types of cancer [1,2,3]. CSCs make up only a relatively small proportion of cancer cells and similar to other stem cells, possess stem-like characteristics [4]. They are also known as cancer-initiating cells because they can readily generate tumours when injected into immunodeficient mice [5]. Under most conditions CSCs are quiescent, but they can be activated to induce self-renewal and to reproduce progenitor cells [2,6]. Studies have shown that although many cancer drugs can effectively eliminate cancer cells, the presence of CSCs will often lead to cancer metastasis and drug resistance, and their survival is responsible for tumour recurrence [1,7]. Clearly, the existence of CSCs poses a unique set of challenges in the treatment of cancers with growing evidence suggesting that eradication of CSCs is a vital overall strategy for developing a successful cancer treatment regime [8]. Thus, by targeting CSCs, some of the limitations of current cancer treatment might be addressed [9]. In the future, cancer treatment regimens may target a combination of cancer cell types including CSCs; however, at present, there is a very limited understanding of the interactions between CSCs and drug treatment.

CSC research is a rapidly evolving field in cancer biology and its application in cancer therapy faces several challenges including CSC heterogeneity and plasticity. These CSC properties make it difficult to treat cancers and assess the outcomes of drug treatment. One problem is the lack of universal CSC markers as observed in single cell analysis [10]. In breast cancer, at least one major cellular marker and two functional assays are frequently used to identify breast CSCs (BCSCs) [11]. Mesenchymal-like CSCs, also known as CD44^+^/CD24^−/low^ CSCs, are relatively well-characterized with high levels of expression of the cell surface marker CD44 and low or no expression of the surface marker, CD24 [12]. Epithelial-like CSCs or ALDH^high^ CSCs are also commonly recognized. These have high aldehyde dehydrogenase (ALDH) activity, which catalyses the oxidation of aldehyde [13]. By using the ALDEFLOUR assay (Stemcell technology), epithelial-like CSCs can be detected by flow cytometry. Additionally, some cancer cells are able to form spheres when cultured in three-dimensional conditions of low nutrition and are considered to be cells with CSC potential. Small tumorspheres have been reported to initiate tumours in murine models [14]. Although some anti-CSC agents are in clinical trials, CSC plasticity remains one of the major obstacles for developing anti-CSC therapies [15]. For example, current studies report that some cancer cells can switch between non-CSC and CSC states [6]. Although this process may be caused by environmental changes and/or epigenetic regulation, the exact mechanisms are still unclear [6]. Thus, while drugs may target existing CSCs, they may induce some non-CSCs to switch to CSC status leading to the regeneration of cancers [15]. Therefore, a drug which could eradicate both non-CSCs and CSCs might be a promising next-generation therapeutic to improve cancer prognostic outcomes. 

The anticancer properties of antiretroviral drugs first drew the attention of clinicians when it was realized that the incidence of AIDS-related cancers was significantly reduced after treating AIDS patients with this class of drugs [16]. To some, this was viewed as a specific effect of virus suppression; however, other researchers believed this outcome was a result of the inhibitory property of endogenous reverse transcriptase derived from long interspersed nucleotide element 1 (LINE-1), a highly mobile transposable element residing in the human genome [17]. Reverse transcriptase (RT) inhibitors are the most widely used antiretroviral agents, and many of them are highly effective at halting HIV amplification [16]. Recently, some RT inhibitors have been evaluated for the treatment of epithelial cancers in cell culture experiments and in murine models [18]; however, whether they can also target CSCs remains unclear. Evidence suggests that cancer treatments that can alter epithelial-to-mesenchymal transition (EMT)-regulatory genes might possibly further influence CSCs [19]. The drug Efavirenz is a commonly used antiretroviral drug that acts by inhibiting the activity of the reverse transcriptase of HIV [20] and has been shown to reverse EMT in cancer cells in culture [21,22]. Here, we focus on the effect of Efavirenz treatment in breast cancer cells and examine whether this drug can be used as a treatment/preventative strategy against CSCs.

## 2. Methods

### 2.1. Cell Cultures and Drug Treatments

For traditional two-dimensional cultures, non-cancerous MCF10A cells and tumorigenic MCF10AT and MCF10CA1α triple-negative breast cancer cells were cultured in DMEM/F-12 medium (Gibco™) with 5% horse serum (Gibco™, Scoresby, VIC, Australia), 10 µg/mL insulin (Sigma-Aldrich, Sydney, NSW, Australia), 20 ng/mL epidermal growth factor (Sigma-Aldrich), 0.5 µg/mL hydrocortisone (Sigma-Aldrich) and 100 ng/mL cholera toxin (List Biological Laboratories, Campbell, CA, USA). MDA-MB-231, a triple-negative breast cancer cell line, and T47D, a luminal type of breast cancer cell line, were cultured in complete DMEM medium (Gibco™, Scoresby, VIC, Australia) with 10% foetal bovine serum (Gibco™, Scoresby, VIC, Australia). All cell lines were purchased from the American Type Culture Collection (ATCC, Manassas, VA, USA). 

Cancer cells were harvested from 2D cultures and were washed with DPBS. Afterward, 20,000 cells were plated on a new ultra-low adherent 6-well plates (Corning^®^, Mulgrave, VIC, Australia) with MammoCult™ tumorsphere culture medium (STEMCELL™ Technologies, Tullamarine, VIC, Australia) for at least 72 h before forming sufficient tumorspheres (formation of a dark centre within tumorsphere is caused by cell death [23]. Efavirenz was added to the tumorsphere culture for a further 6 days and the medium containing Efavirenz was changed every 3 days. Tumorsphere images were taken on the seventh day. The EC50 of Efavirenz was determined in a previous publication [22]. 

For drug treatment experiments, Efavirenz (Sigma-Aldrich, Sydney, NSW, Australia) was mixed with the cell culture medium, and the pre-seeded cells were incubated with the drug-containing medium for four days. An equivalent amount of dimethyl sulfoxide (DMSO) (Sigma-Aldrich, Sydney, NSW, Australia) was added to the medium for parallel negative controls. The incubating medium was replaced with fresh drug or DMSO-containing medium every 48 h.

Western blotting result images were captured with an ImageQuant LAS 4000 biomolecular imager (GE healthcare). The intensity of each band was measured by ImageJ freeware and calculated with Microsoft Excel. Triplicates Western blotting results were collected and p-values were calculated by using a paired two-tailed Student’s *t*-test.

### 2.2. XTT Cell Viability Assay 

A 2,3-Bis-(2-methoxy-4-nitro-5-sulfophenyl)-2H-tetrazolium-carboxanilide (XTT) assay (Cell Signaling Technology^®^, Arundel, QLD, Australia) was used to examine cell viability. After mixing the XTT reagent and electron-coupling solution (50:1 ratio), 50 μL of the complete XTT detection reagent was added to each utilized well of 96-well plates. The plates were then incubated for 4 h in a 37 °C incubator to allow for the enzyme reaction to take place. Finally, the absorbance of each well was measured at 450 nm using an iMark ELISA reader (Bio-Rad, Gladesville, NSW, Australia).

### 2.3. Immunofluorescence (IF)

Before treatment, cells were seeded on 12 mm Poly-D-lysine-coated glass coverslips (Neuvitro, Cat# GG-12-1.5-PDL) in a 24-well plate for one day until the cells attached to the coverslip surface. For IF staining, cells were fixed on coverslips using immunofluorescence fixation solution (4% formaldehyde, Sigma-Aldrich). Afterwards, cells were permeabilised by 0.25% Triton X-100 (Sigma-Aldrich) followed by incubation with blocking buffer. The permeabilised cells were then incubated with fluorescent conjugates of phalloidin. The coverslip with stained cells was then mounted with ProLong™ Gold/Diamond Antifade Mountant and the nuclear stain, DAPI (Molecular Probes^®^, Scoresby, VIC, Australia). Finally, the coverslips were visualised using a Leica SP5 confocal microscope. 

### 2.4. Cancer Stem Cell (CSC) Flow Cytometry

An ALDEFLUOR^®^ kit was used to identify epithelial-like CSCs with high aldehyde dehydrogenase (ALDH) activity. The samples were incubated with pre-mixed, activated ALDEFLUOR^®^ substrate in ALDEFLUOR^®^ buffer to allow for endogenous ALDH to digest the substrates. In the meantime, the same number of cells was incubated with ALDEFLUOR^®^ substrate under similar conditions to the test cells apart from the addition of N, N-diethylaminobenzaldehyde (DEAB) to block the endogenous ALDH function. After incubation, the test sample and control cells were detected using flow cytometry and the data were collected and analysed by Flowjo™ software version 10.5. The high ALDH activity cells were gated based on the ALDH inhibition control. Cells brighter than the ALDH inhibition control were considered to be high ALDH activity cells. 

To identify the expression of the cell surface markers CD44 and CD24, the harvested cells were stained with an anti-CD44 antibody conjugated with PE fluorescence (1:100 dilution, BD Pharmingen, San Diego, CA, USA) and anti-CD24 antibody conjugated with PE-Cy7™ or Alexa Fluor^®^ 647 (1:50 dilution, BD Pharmingen). Samples were incubated with the target antibodies at 4 °C in darkness for 30 min, followed by gentle washing steps. After a complete residue dye wash, samples were analysed by a BD Fortesssa flow cytometry (BD Biosciences, San Diego, CA, USA). The data were collected and further analysed by Flowjo software. 

### 2.5. mRNA-Seq Gene Expression Profiling

Total RNA was extracted from samples using an RNAqueous™ Total RNA Isolation kit (ThermoFisher Scientific, Scoresby, VIC, Australia). For each treatment three high-quality (evaluated with an Agilent 2100 Bioanalyzer) RNA samples were then processed through an RNA sequencing process. The experimental design was a two-factor ANOVA design with 12 samples including triple-negative cell lines (MCF10A, MCF10AT, MCF10CA1α and MDA-MB-231) treated with Efavirenz and DMSO. In total, 1296 genes had raw *p*-values < 0.05 for the Efavirenz versus DMSO contrast. For the Efavirenz versus DMSO treatment contrast, 198 genes passed a false discovery threshold of 0.1625 using Storey’s q-value test implemented in the R qvalue library.

### 2.6. microRNA Expression Taqman Assays and microRNA Profiling by Microarrays

TaqMan^®^ Advanced microRNA assay (ThermoFisher Scientific, Scoresby, VIC, Australia) was used for miRNA-RT-PCR. Total RNA was prepared with the mirVana™miRNA Isolation kit (ThermoFisher Scientific) to enrich small RNAs. The cDNA was synthesised by the TaqMan^®^ Advanced miRNA cDNA Synthesis Kit (ThermoFisher Scientific). Then, 5 μL of 1 in 10 diluted cDNA templates was mixed with 10 μL TaqMan^®^ Fast Advanced Master Mix (2×), 1 μL TaqMan^®^ Advanced miRNA assay (20×) and 4 μL RNase-free water. The mixture was then transferred into a 96-well PCR plate and measured by a StepOnePlus™RT-PCR instrument (Applied Biosystems, Scoresby, VIC, Australia). The ExpressionSuite™ software was used for analysing the results by comparing the relative quantification ΔΔCt values among samples. All the samples included four technical replicates to ensure consistency.

For genome-wide microRNA expression profiling by microarrays, total RNA integrity was evaluated with an Agilent 2100 Bioanalyzer. Approximately 20 ng RNA was labelled with Cy3-conjugated dCTP (Amersham) using the PrimeScript (Takara) reverse transcriptase. Labelled cDNA was hybridized for 16 h at 42 °C to Roche NimbleGen Human Whole-Genome 12-plex arrays according to the manufacturer’s protocol. The arrays were analysed with an Axon GenePix 4000B scanner and associated software (Molecular Devices). miRNA expression levels were calculated with NimbleScan Version 2.4 (Roche NimbleGen, Inc., Madison, WI, USA).

The bioinformatics analysis pipeline was identical to that described in [24].

## 3. Results

### 3.1. Changes in Cancer Stem Cell Regulators/Indicators Observed in Efavirenz-Treated Breast Cancer Cells

The use of Efavirenz as a potential cancer treatment for triple-negative breast cancers was previously explored by our group in various breast cancer cell lines [22]. These studies showed that at EC50 concentrations, Efavirenz significantly reduced cell viability in MCF10AT, MCF10CA1α, MDA-MB-231 and T47D breast cancer cell lines (Figure S1B) and therefore, similar treatment conditions were used in subsequent CSC experiments undertaken in this study [22]. Prior to embarking upon CSC experiments, bright-field microscopy was used to confirm morphological variations under drug treatment conditions. Changes in cell morphology were clearly visible in all tested Efavirenz-treated breast cancer cell lines but not in the non-cancerous MCF10A cell line (Figure S1A). In contrast to untreated breast cancer cells, which showed indistinct cell borders, numerous drug-treated breast cancer cells displayed cell death phenotypes or distinct cell borders with flattened patterns or neuron-like projections, which are considered to be traits of cell differentiation [22]. These results were consistent with the morphological changes shown in other types of cancers treated with Efavirenz [17,22]. 

**Figure 1:**
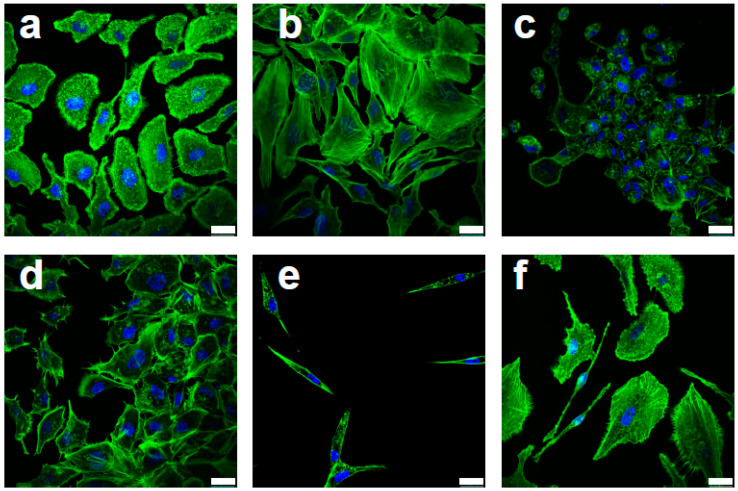
Efavirenz-induced morphological changes in F-actin distribution for MCF10AT breast cancer cells. (**a**) Untreated control cells. (**b**–**f**) Different cell morphologies were observed in Efavirenz-treated MCF10AT cells: (**b**) flattened and angular morphology, (**c**) cell death phenotype, (**d**) neuron-like cell projection, (**e**) quiescent phenotype and (**f**) migrating cells presenting filopodia and clear direction. Cells were stained with Phalloidin (green, for F-actin detection) and DAPI (blue, for nucleus detection). Scale bar: 25 μm. These images were produced by Leica SP5 confocal microscope: objectives lenses—63×/1.40 (oil).

**Figure 2:**
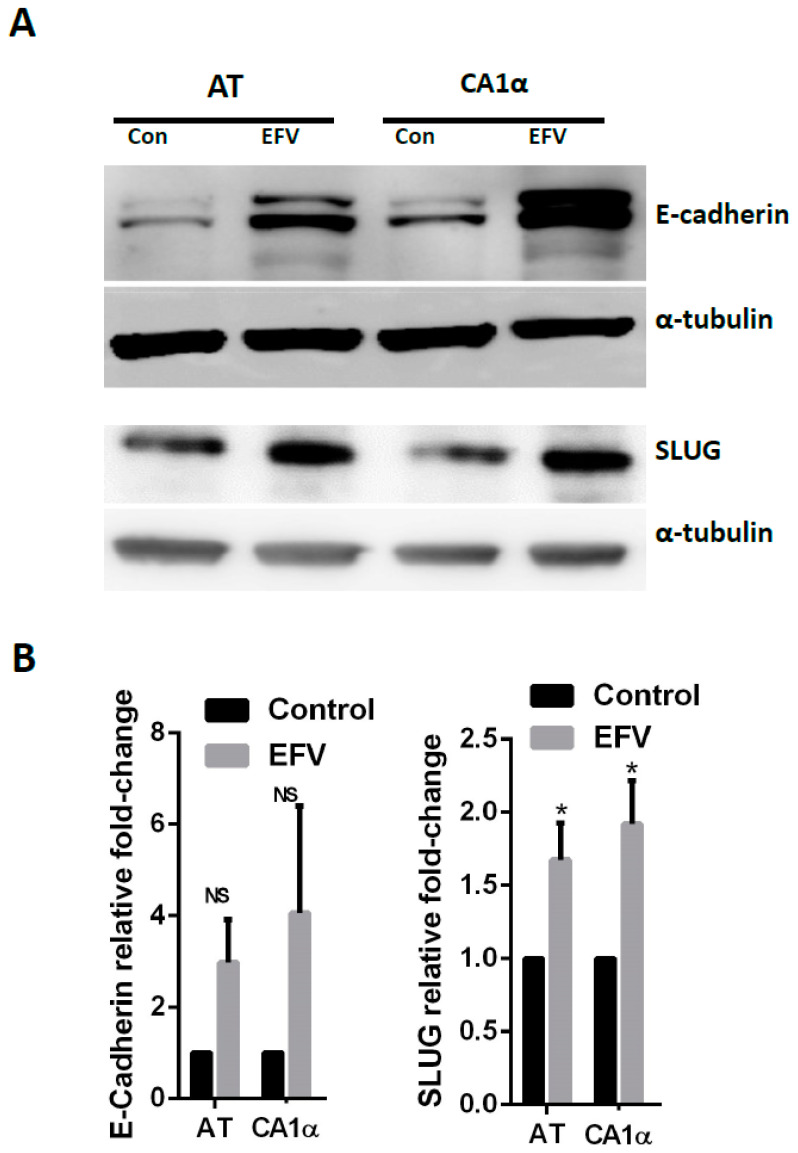
Efavirenz-induced molecular change in breast cancer cells. (**A**) The expression of E-cadherin (epithelial marker) increased in Efavirenz-treated MCF10AT and MCF10CA1α cells. The expression of SLUG (mesenchymal marker) also increased in drug-treated breast cancer cells. (**B**) Normalized fold change of E-Cadherin and SLUG. Error bars: ± SD, *n* = 3. (paired Student’s *t*-test, * *p* < 0.05).

Characteristic features of malignant cells were also seen in Efavirenz-treated breast cancer cells, especially in the MCF10AT cells. Based on their cytoskeletal actin distribution as detected by F-actin labelling (Phalloidin staining), most of the drug-treated cells displayed epithelial phenotypes (Figure 1b,d) or cell death phenotypes (Figure 1c) indicating drug-induced anticancer effects. However, some of the cells displayed quiescent phenotypes (Figure 1e), which are usually associated with CSCs [6], while other cells displayed observable migratory behaviour (Figure 1f), which is synonymous with cancer metastasis [25]. Comparable features were also observed in MCF10CA1α and MDA-MB-231 cell lines (Figure S1C). Western blot analysis was consistent with the immunofluorescence results and showed that, after Efavirenz treatment, simultaneous upregulation of E-cadherin, (an epithelial marker and CSC regulator) [26], and SLUG and Fibronectin (mesenchymal markers and potential CSC activators) [27,28] occurred (Figure 2 and Figure S4). These markers were also used to examine morphological changes upon Efavirenz treatment (Figure S2). Typically, these malignant phenotypes are linked to unfavourable prognostic outcomes and most of them can be linked to CSCs.

**Figure 3:**
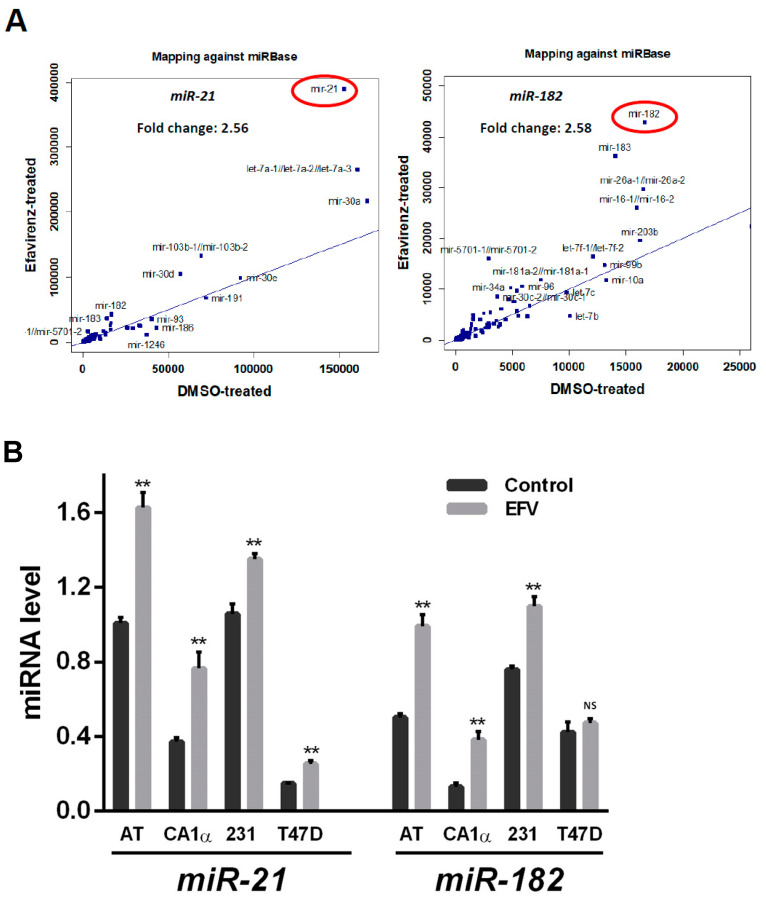
miRNA changes in Efavirenz-treated breast cancer cells. (**A**) Scatter plot based on microarray analysis. Efavirenz-treated T47D cells versus untreated-control T47D cells (DMSO). Blue symbols denote miRNAs. *miR-21* and *miR-182* have greater fold changes than other miRNAs. (**B**) microRNA RT-qPCR analysis for *miR-21* and *miR-182* in Efavirenz-treated and untreated MCF10AT, MCF10CA1α, MDA-MB-231 and T47D cells. *miR-21* and *miR-182* qPCR results were normalized with an internal control miR-423. Error bars: ± SD, *n* = 3. (paired Student’s *t*-test, ** *p* <0.01).

Experiments involving RNA and microRNA expression were performed in Efavirenz-treated triple-negative breast cancer cell lines, MCF10AT, MCF10CA1α and MDA-MB-231, and the non-cancerous cell line, MCF10A. Upon analysis, levels of several CSC-related genes were significantly altered by Efavirenz treatment and are displayed in Table 1. These included CSC-related genes such as *MED8* (mediator complex subunit 8), *DMXL2* (Dmx-like protein 2) and *PROCR* (protein C receptor), all of which increased after Efavirenz treatment; whereas the expression of other CSC-related genes including *CHMP4B* (charged multivesicular body protein 4B), *ACSL3* (acyl-CoA synthetase long-chain family member 3), *FASN* (fatty acid synthase) and *SCD* (stearoyl-CoA desaturase) decreased. A reduction in ACSL3, FASN and SCD mRNA expression has been observed across the different breast cancer cell lines and linked with fatty acid metabolism-associated genes [22]. Additionally, small RNA expression profiling by microarray (Table 2) also gave rise to an upregulation of *miR-21*, a reported CSC marker [29], and *miR-182*, a CSC suppressor [30,31,32] in Efavirenz-treated T47D cells (Figure 3A). microRNA RT-qPCR analysis across different breast cancer cell lines (Figure 3B) indicate that this trend is maintained with *miR-21* and *miR-182*. The non-cancerous cell line, MCF10A showed expression of both *miR-21* and *miR-182,* which did not change upon Efavirenz treatment. On the whole, these results reflect the diverse genetic changes observed and further emphasise the complexity of Efavirenz-induced CSC regulation. 

### 3.2. Breast CSC Population Are Altered by Efavirenz Treatment

**Figure 4:**
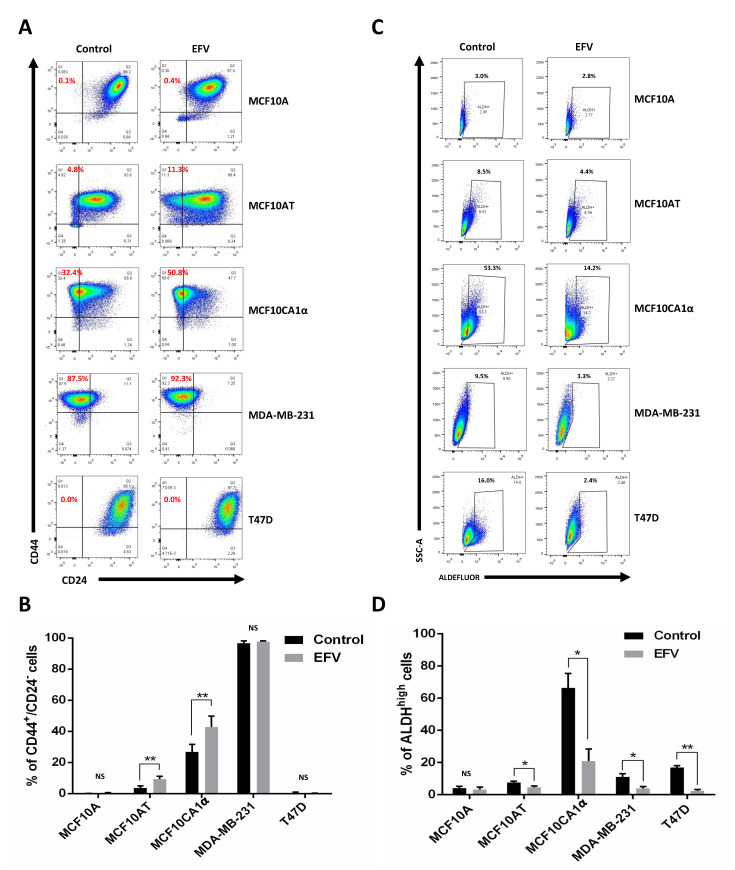
Effects of *Efavirenz treatment on mesenchymal* and *epithelial CSC levels in breast cancer cell lines.* (**A**) Flow cytometry plots for CD44^+^/CD24^-^ mesenchymal-like CSCs cells detected in untreated and EFV-treated cells. (**B**) Percent of mesenchymal-like CSCs cells presented in untreated and EFV-treated cells. (**C**) Flow cytometry plots for ALDH^high^ epithelial-like CSCs cells detected in untreated and EFV-treated cells. (**D**) Percent of the epithelial-like CSCs presented in EFV-treated and untreated cancer cells. Error bars: ± SD, *n* = 3. (paired Student’s *t*-test, * *p* < 0.05; ** *p* <0.01).

To determine the effects of Efavirenz in BCSCs, epithelial-like CSCs were identified using the ALDEFLUOR^®^ kit and mesenchymal-like CSCs were detected by CD44/CD24 staining. The proportion of epithelial-like CSCs was reduced by at least 1.5-fold after Efavirenz treatment in all tested breast cancer cell lines with very little reduction observed in the non-cancerous MCF10A cell line (Figure 4C,D). The percentage of the epithelial-like CSCs decreased as described in Table 3. Notably, epithelial-like CSCs in Efavirenz-treated MCF10CA1α and MDA-MB-231 cells decreased by 3-fold compared to their untreated controls. T47D cells, a luminal-type breast cancer cell line, showed a 7-fold decrease in its epithelial-like CSC population after Efavirenz treatment. All these cancer cell lines displayed significant differences between Efavirenz-treated cells and DMSO-treated control cells, whereas changes in non-cancerous MCF10A cells were not statistically significant (*p*-value = 0.233) (Figure 4C). These data demonstrated that the proportion of epithelial-like CSCs can be reduced by Efavirenz treatment.

In addition to epithelial-like CSCs, mesenchymal-like CSC levels were also monitored as a result of Efavirenz treatment. The results of CD44/CD24 staining in two of the triple-negative breast cancer cell lines tested (MCF10AT and MCF10CA1α) indicated an overall increase in mesenchymal-like CSC population after treatment with Efavirenz (Figure 4A,B), opposite to the results obtained for epithelial-like CSCs. The degree of change varied from no change up to a ~2.5-fold increase in the fraction of the mesenchymal-like CSC population. Very few mesenchymal-like CSCs were detected in the MCF10A (non-cancerous control) and T47D cells (luminal-type breast cancer), while high mesenchymal-like CSC population levels were maintained in MDA-MB-231 cells. Statistical analysis of these results showed significant differences in the population of mesenchymal-like CSCs under Efavirenz-treated and untreated conditions in two of the cell lines tested, indicating that the effects of Efavirenz treatment on CD44^+^/CD24^−^ mesenchymal-like CSC populations are cell line dependent.

To explore the relationship between LINE-1 and Efavirenz-treated CSCs, LINE-1 inhibition by short hairpin RNA (shRNA) was employed to confirm the mesenchymal-CSC results. A pUTR plasmid (Figure S2A), encoding an shRNA sequence targeting the LINE-1 promoter, was transfected into MCF10AT and MCF10CA1α cells in order to inhibit LINE-1 expression. The non-functional empty vector (pSM2 plasmid, Figure S2A) was transfected as a negative control for the pUTR plasmid. Although the shRNA inhibition results were not straightforward to interpret owing to the extremely fast cell proliferation rates of the cancer cells, there was an increase in the mesenchymal-like CSC population upon transfection with the pUTR plasmid (Figure S2B). The percentage of the mesenchymal-like CSCs was 0.5% in the MCF10AT-pSM2 cells and 9.3% in the MCF10AT-pUTR cells, whereas it was 14.5% in the MCF10CA1α-pSM2 and 27.8% in the MCF10CA1α-pUTR cells (Figure S2B). Both MCF10AT and MCF10CA1α cells with partial LINE-1 silencing displayed greater numbers of mesenchymal-like CSCs compared to their controls. Therefore, LINE-1 inhibition through shRNA increased the mesenchymal-like CSC population in the MCF10AT and MCF10CA1α cell lines was consistent with the previously observed results arising from Efavirenz treatment (Figure 4A,B).

### 3.3. Efavirenz Can Effectively Reduce Functional Breast CSCs

**Figure 5:**
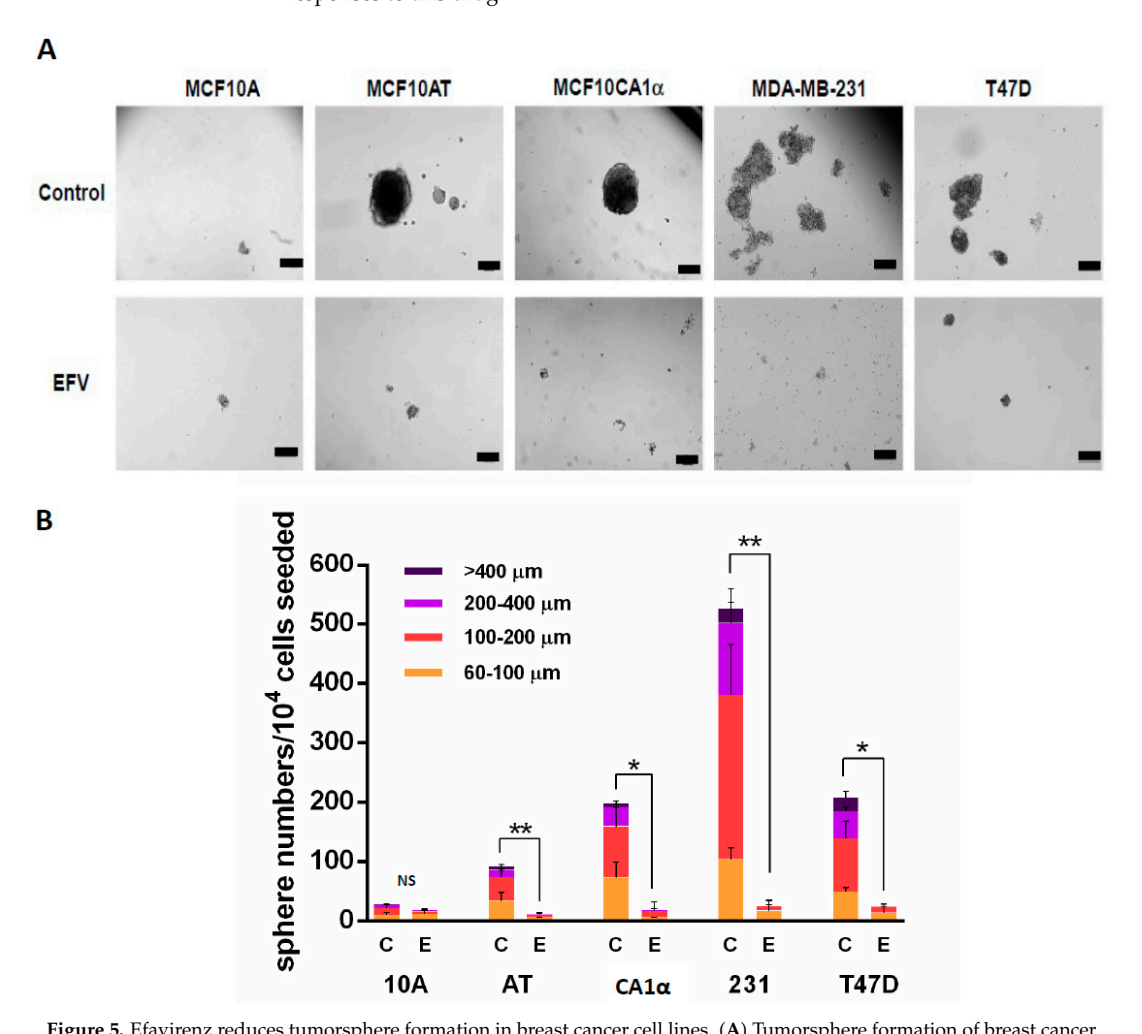
Efavirenz reduces tumorsphere formation in breast cancer cell lines. (**A**) Tumorsphere formation of breast cancer MCF10AT, MCF10CA1α, MDA-MB-231 and T47D cells were disrupted by Efavirenz treatment based on their EC50 values described in our previous study [22]. (**B**) Numbers of tumorspheres formed in untreated and Efavirenz-treated breast cancer cells. Any clump smaller than 60 μm is not considered as a tumorsphere. Two-tailed, paired Student’s *t*-test was conducted for statistical analysis, and the total cell numbers are compared between untreated and Efavirenz-treated conditions. (paired Student’s *t*-test, * *p* < 0.05; ** *p* <0.01). Error bars: ± SD, *n* = 3. C: untreated control cells, E: Efavirenz-treated cells. Scale bar: 200 μm.

After observing changes in BCSC levels as a result of Efavirenz treatment, it was of interest to further explore the role of Efavirenz and BCSCs in cancer development. Ideally, measuring the effects of Efavirenz in a CSC-only population would be a better way of monitoring this population; however, maintaining CSCs in traditional two-dimensional culture conditions is challenging [6]. Therefore, growing cancer cells in the MammoCult™ medium (STEMCELL Technologies) was used as an alternative strategy for undertaking CSC experiments. Cultivation of cells in MammoCult™ Medium is a recognised three-dimensional culture method specifically used for breast cancers to enrich cells with CSC-forming ability [40]. Tumorspheres formed in the MammoCult™ medium can subsequently initiate tumours when injected into immunodeficient mice, indicating that the cancer cells that can grow and form tumorspheres in the MammoCult™ medium are functional CSCs. Interestingly, the relative numbers of tumorspheres formed for each cell line mostly correlated with the relative malignancy of the cancer cell lines [41]. The most malignant cell line, MDA-MB-231, formed the largest number of tumorspheres compared with other cell lines, while the less-invasive MCF10AT cells produced the lowest number of tumorspheres (Figure 5A). These spheres were further exposed to Efavirenz in order to examine their responses to this drug.

Treatment of tumorspheres with Efavirenz resulted in changes in the tumorsphere structures (from tight to loose) (Figure 5A), while also causing a reduction in the size and number of the spheres (Figure 5B). After Efavirenz treatment, the total number of tumorspheres declined from 92.3 ± 16.2 to 11.0 ± 6.2 in MCF10AT, from 198.0 ± 56.8 to 18.3 ± 18.0 in MCF10CA1α, from 525.0 ± 25.7 to 35.2 ± 25.4 in MDA-MB-231 and from 158.0 ± 36.9 to 10.8 ± 4.5 in T47D cells. There were significant differences in the total number of tumorspheres in Efavirenz-treated and untreated cancer cell lines but not in the non-cancerous MCF10A cell line (*p*-value = 0.191633). Notably, after Efavirenz treatment, the tumorsphere number was roughly 20-fold less than that of the controls in MDA-MB-231, 15-fold less in T47D, 11-fold less in the MCF10CA1α and 8-fold less in MCF10AT cells. These data strongly suggested that the functional CSCs can be dramatically reduced by Efavirenz treatment, thus indicating that Efavirenz has an impact on both non-CSCs and CSCs.

## 4. Discussion 

CSCs are believed to be one of the main drivers of cancer metastasis and have been linked to resistance and to patient relapse in conventional drug and radiation therapies [1]. They are, therefore, an attractive target for improving the prognostic outcomes of cancer patients. In this study, several commonly used methods for identifying and analysing BCSC were utilised to further understand the effects of the Efavirenz on BCSCs. This study adds to an emerging field of repurposing antiviral inhibitors to treat malignant cancers [42] and represents one of the first studies directly focusing on the use of an antiretroviral reverse transcriptase inhibitor drug to target CSCs.

**Figure 6:**
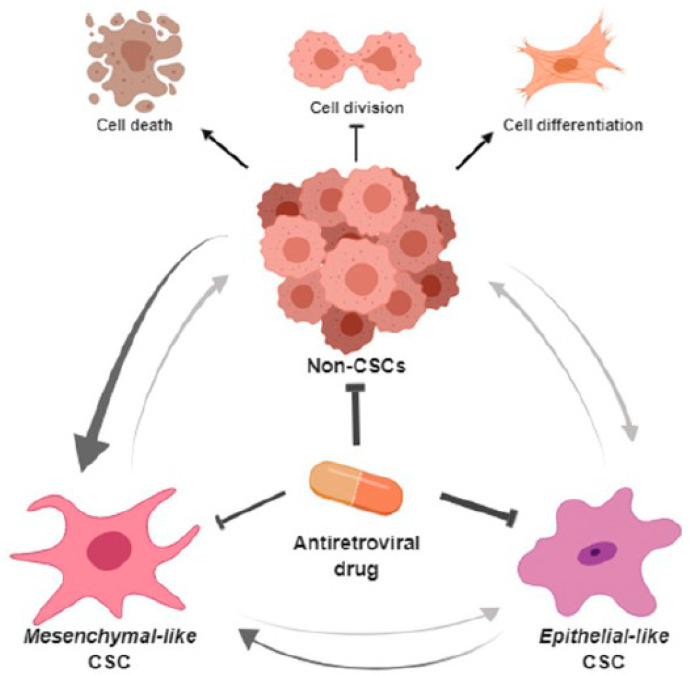
Putative roles of Efavirenz in breast cancer treatment. Efavirenz may antagonise the progress of breast cancers by targeting both CSCs and non-CSCs. It can promote cell differentiation in undifferentiated cancer cells, can suppress cancer cell division and can induce cancer cell death. Although efavirenz has the ability to reduce the epithelial-like CSCs, mesenchymal-like CSCs are more resistant to antiretroviral drugs compared with other types of breast cancer cells. Furthermore, cancer cells can potentially switch their status between CSCs and non-CSCs because of their plasticity. Breast cancer cells tend to have a preference for maintaining and/or increasing their mesenchymal-like CSC status under antiretroviral drug treatment condition. (Schematic diagrams were created by bioRender online software).

The most striking finding of this study is the ability of Efavirenz to affect the numbers of both general cancer cells and CSCs in breast cancers as summarised in Figure 6. This schematic diagram outlines the interplay between CSCs (mesenchymal-like and epithelial-like) and non-CSCs and their response to Efavirenz treatment. In our experiments, distinctive types of breast cancer cell lines displayed very different CSC profiles. For instance, MCF10CA1 cells showed a high level of epithelial-like CSCs, whereas in MCF10CA1α cells, a very high level of mesenchymal-like CSCs was observed. These cell lines were found to respond differently to Efavirenz treatment. ALDH^high^ epithelial-like CSCs were significantly decreased after Efavirenz treatment, whereas in some breast cancer cell lines CD44^+^/CD24^−^ mesenchymal-like CSCs showed an increase. These results are internally consistent with some Efavirenz-treated breast cancer cell lines displaying epithelial phenotypes, whereas others displayed mesenchymal and CSC markers typically associated with unfavourable prognostic outcomes. These findings highlight the complexity, heterogeneity and plasticity of breast cancers and indicate that different types of CSCs may respond differently to particular chemotherapeutics. Our results suggest that mesenchymal-like CSCs are more resistant to Efavirenz than other BCSC types with the possibility that LINE-1 inhibition may promote EMT and/or the conversion of non-CSCs to a mesenchymal-like CSC status. This raises an intriguing question relating to the overall benefits of treating breast cancers with antiviral drugs and whether the potential for such a therapy is limited? The answer no doubt will become clearer when studies are extended into animal and patient models.

Tumorsphere formation is widely used to analyse the self-renewal capability of CSCs, and in this study, the tumorsphere CSC functional assay demonstrated that Efavirenz treatment reduced the numbers of CSCs. Traditional in vitro two-dimensional culture conditions do not replicate the physiological tumour microenvironment [43] raising the question of how well such experimental conditions actually mimic the native environment of CSCs. Even though MammoCult three-dimensional cultures may partially address this problem, little is known about how many real CSCs can accumulate within this culture. Furthermore, the tumorsphere experiments in this study were performed on a small scale with only a few tumorspheres remaining after Efavirenz treatment. Nonetheless, this pilot study provides an alternative methodology to demonstrate that an antiviral drug can influence numbers of CSCs. More extensive tumorsphere studies need to be undertaken before more quantitative conclusions can be made using this technique. 

Analysis of the mRNA-Seq data and previous microRNA expression profiling data provides a potential insight into LINE-1 repression-promoted anticancer pathways. A notable observation is the upregulation of the microRNA *let-7a* in LINE-1 silenced T47D cells [24] and the downregulation of SCD in Efavirenz-treated breast cancer cells. SCD is recognised to facilitate cancer stemness [39] and has been shown to be regulated by *let-7a* [44]. Although some of the fold changes in Table 2 are small and further verification will be required to confirm the significance of these changes in gene expression, the greatest change observed was for SCD, indicating that inhibition of SCD expression via upregulation of *let-7a* may lead to a reduction of CSC numbers. Upregulation of the microRNAs *miR-21* and *miR-182* was also observed upon Efavirenz treatment. miR-182 is a known tumour suppressor with administered miR-182-based therapies reducing tumour burden and increasing animal survival [35]. An increase in *miR-182* levels after treatment with Efavirenz suggests a potential benefit with this strategy. In addition to changes in micoRNAs, Efavirenz treatment has also been linked to cancer regulatory pathways including the cannabinoid system [45], oxidative stress metabolism [46], the Type-I interferon response [47] and fatty acid metabolism [22]. As an upstream controller, it is most likely that Efavirenz targets cancers by regulating multiple tumorigenic pathways. However, more targeted studies are needed to further dissect these pathways before their roles in CSCs and non-CSCs can be definitively mapped.

CSC heterogeneity and plasticity are major challenges in the CSC research field, with CSC maintenance and the CSC-enrichment methods proving particularly difficult to control, making it particularly difficult to assess molecular changes in drug-treated functional CSCs. In addition to these issues, an overreliance on select CSC markers should be carefully considered. For instance, *miR-21* has often been ascribed as a marker for CSCs, and in this study, the observed increase of *miR-21* upon Efavirenz treatment is not consistent with an expected reduction in CSC levels. It is notable that elevated *miR-21* levels have been observed across numerous non-neoplastic diseases and its usefulness as a specific cancer biomarker is debatable [48]. Therefore, caution should be exercised when relying on specific markers when determining cell status, and supporting evidence is always recommended to validate results. CSC research is still a relatively underdeveloped field, and many hypotheses and assumptions need to be established and tested. Advances in this field will facilitate the establishment of more robust CSC methodologies and will lay the platform for expanding future studies that encompass animal models and cancer patient clinical samples.

## 5. Conclusions

This study highlights the various complexities and challenges surrounding the therapeutic inhibition of targets involved in breast CSCs. It is clear that, in order to evaluate the effects of therapeutics such as Efavirenz on breast CSCs, a deeper understanding of stem cell signalling networks and the interplay between various pathways will be required. As has been observed with several studies [1,15,19,43], many strategies designed to target CSCs have been met with limited degrees of success, and an alternative future strategy may be to adopt a combination of therapeutic options targeting diverse pathways associated with CSCs. We have shown that one of these pathways involves transposable elements in the DNA (LINE-1) and in what represents a novel mode of action, we have shown by the use of the antiviral drug Efavirenz, it is possible to not only eliminate primary breast cancer cells, but also promote differentiation of breast CSCs.

## Figures and Tables

**Table 1:** CSC-related genes involved in Efavirenz-induced cancer responses for triple-negative breast cancer cell lines (MCF10A, MCF10AT, MCF10CA1α and MDA-MB-231).

Gene Names	Fold Change	P-Value	References
PROCR	1.777661	7.99 × 10^−4^	[33]
MED8	1.31992	9.71 × 10^−4^	[34]
DMXL2	1.161448	3.93 × 10^−3^	[35,36]
CHMP4B	−1.33233	1.85 × 10^−4^	[37]
ACSL3	−1.73666	3.04 × 10^−4^	[38]
FASN	−1.75391	2.82 × 10^−4^	[39]
SCD	−4.34746	2.48 × 10^−3^	[39]

**Table 2:** Target microRNA sequences.

Mature miRNA Sequence of the Target miRNAs used in microRNA qRT-PCR Target microRNA	Assay ID	Mature miRNA Sequence
miR-21	rno481342_mir	UAGCUUAUCAGACUGAUGUUGA
miR-182	477935_mir	UUUGGCAAUGGUAGAACUCACACU
miR-423	478090_mir	UGAGGGGCAGAGAGCGAGACUUU

**Table 3:** Percentage of epithelial-like/mesenchymal-like CSCs in Efavirenz-treated and untreated breast cells.

		MCF10A	MCF10AT	MCF10CA1α	MDA-MB-231	T47D
Epithelial-like CSCs	Control	4.08 ± 1.17%	7.56 ± 0.71%	66.30 ± 8.97%	11.06 ± 1.96%	16.76 ± 1.17%
	EFV	3.13 ± 1.50%	4.53 ± 0.79% *	20.76 ± 7.51% *	3.87 ± 1.05% *	2.32 ± 0.86% **
Mesenchymal-like CSCs	Control	0.05 ± 0.03%	3.66 ± 1.14%	26.77 ± 4.01%	96.67 ± 1.25%	0.44 ± 0.41%
	EFV	0.38 ± 0.30%	9.30 ± 1.56% **	42.90 ± 5.60% **	98.33 ± 0.47%	0.14 ± 0.11%

(paired Student’s *t*-test, * *p* < 0.05; ** *p* <0.01).

## Data Availability

The data presented in this study are available in the article or supplementary files.

## Supplementary Materials

The following are available online at https://www.mdpi.com/article/10.3390/cancers13246232/s1, Figure S1: Cell viability and morphological changes at EC50 concentrations of Efavirenz in breast cancer cell lines., Figure S2: Cell morphological changes in Efavirenz-treated MCF10AT breast cancer cells., Figure S3: The mesenchymal-like CSCs in MCF10AT-pSM2, MCF10AT-pUTR, MCF10CA1α-pSM2, MCF10CA1α-pUTR cells., Figure S4: Original E-cadherin, SLUG, and Fibronectin Western blotting re-sults with statistical tests.
