# Supplementary material for: The Antiviral Drug Efavirenz in Breast Cancer Stem Cell Therapy
Source: Cancers (Basel). 2021 Dec 11;13(24):6232. doi: 10.3390/cancers13246232 (PMC8699628; doi:10.3390/cancers13246232)
Supplement: Supplementary file 1 [file cancers-13-06232-s001.zip › cancers-1428398-supplementary.pdf]

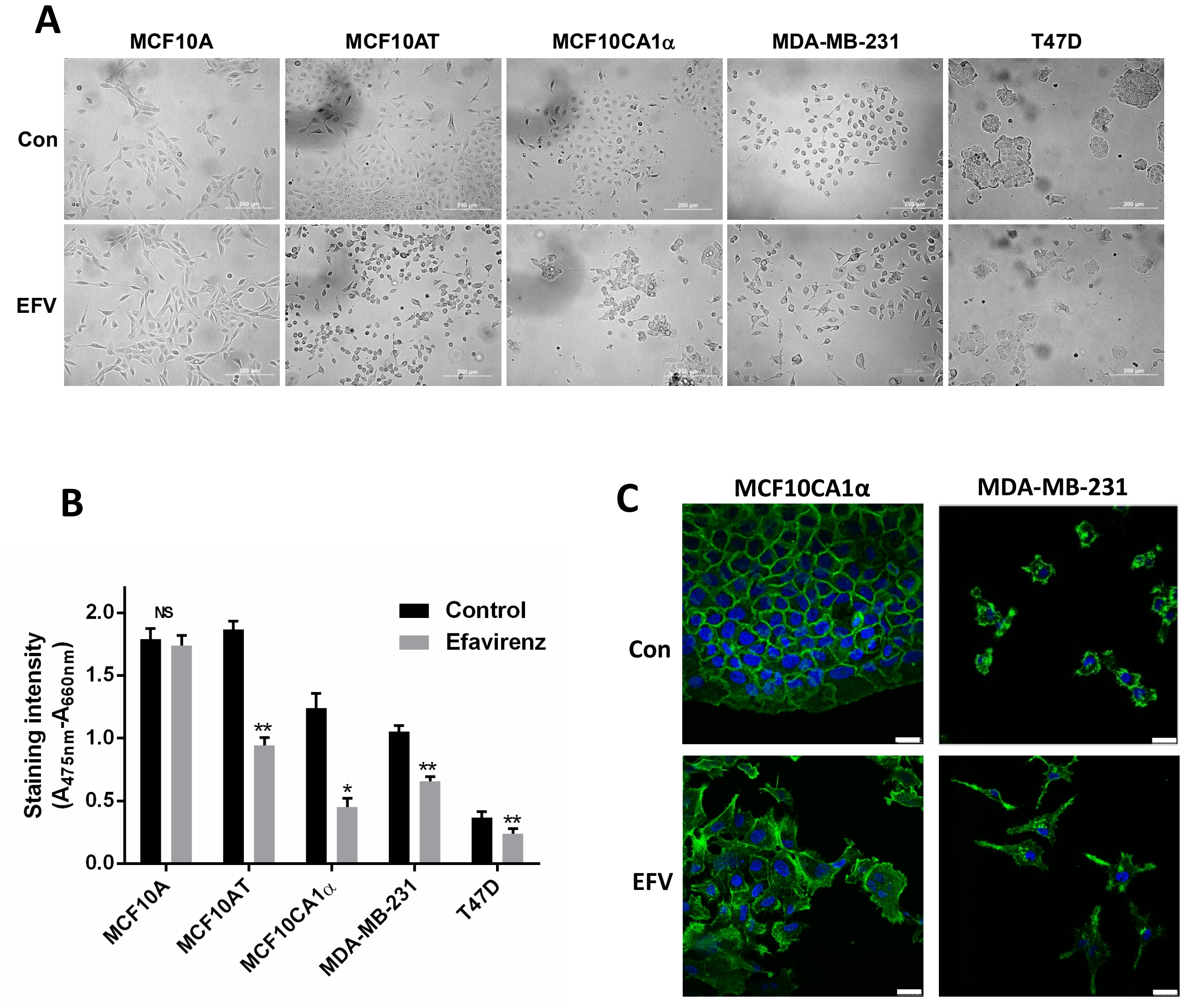

Figure S1: Cell viability and morphological changes at EC50 concentrations of Efavirenz in breast cancer cell lines

A Cell morphologies under untreated- and Efavirenz-treated conditions. Scale bar: 200 μm. These images were produced by Olympus IX71 inverted bright-field/fluorescence microscope: objectives lenses — 20x. **B** The relative cell viability (using XTT cell viability assay) in untreated- and Efavirenz-treated breast cancer cells including MCF10AT, MCF10CA1α, MDA-MB-231, and T47D in 96-hour treatment time point [22]. Efavirenz is toxic to all these tested cells and significantly reduces breast cancer cell viabilities. Error bars: ±SD, n = 3. (paired Student's t-test, * p < 0.05; ** p <0.01). **C** Efavirenz-induced morphological changes in F-actin distribution for MCF10CA1α and MDA-MB-231 breast cancer cells. F-actin was stained by Phalloidin (Green) and nucleus was stained by DAPI (Dark blue). Scale bar: 25 μm. These images were produced by Leica SP5 confocal microscope: objectives lenses—63x/1.40 (oil).

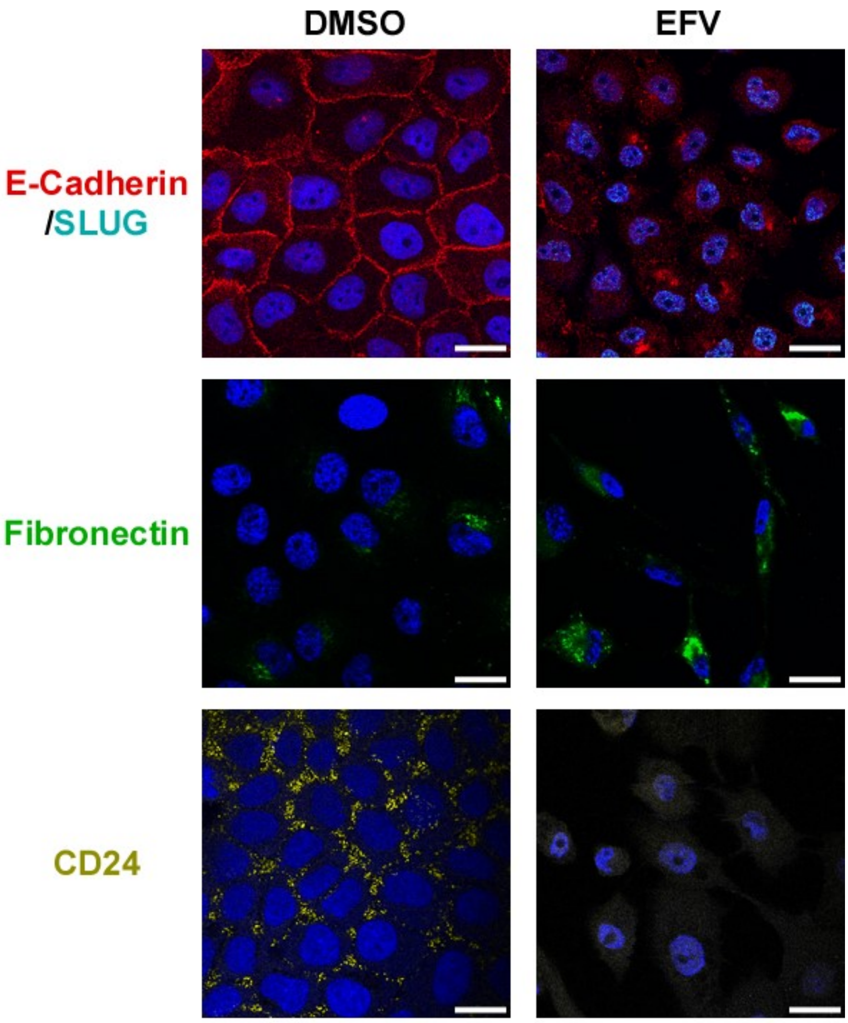

Figure S2: Cell morphological changes in Efavirenz-treated MCF10AT breast cancer cells

Efavirenz-induced morphological changes in E-cadherin (Red), SLUG (Aqua), Fibronectin (Green), and CD24 (Yellow) distribution for MCF10AT breast cancer cells. Nucleus was stained by DAPI (Dark blue). Scale bar: 25 µm. These images were produced by Leica SP5 confocal microscope: objectives lenses— 63x/1.40 (oil).

A **pSM2:**

Sense (mir5') UUCUCCGAACGUGUCACGUdTdT

Anti-sense (mir 3') ACGUGACACGUUCGGAGAAdTdT

pUTR:

Sense (mir 5') TTTACCTAAGCAAGCCTGGGC

Anti-sense (mir 3') TGCCCAGGCTTGCTTAGGTAAA

B

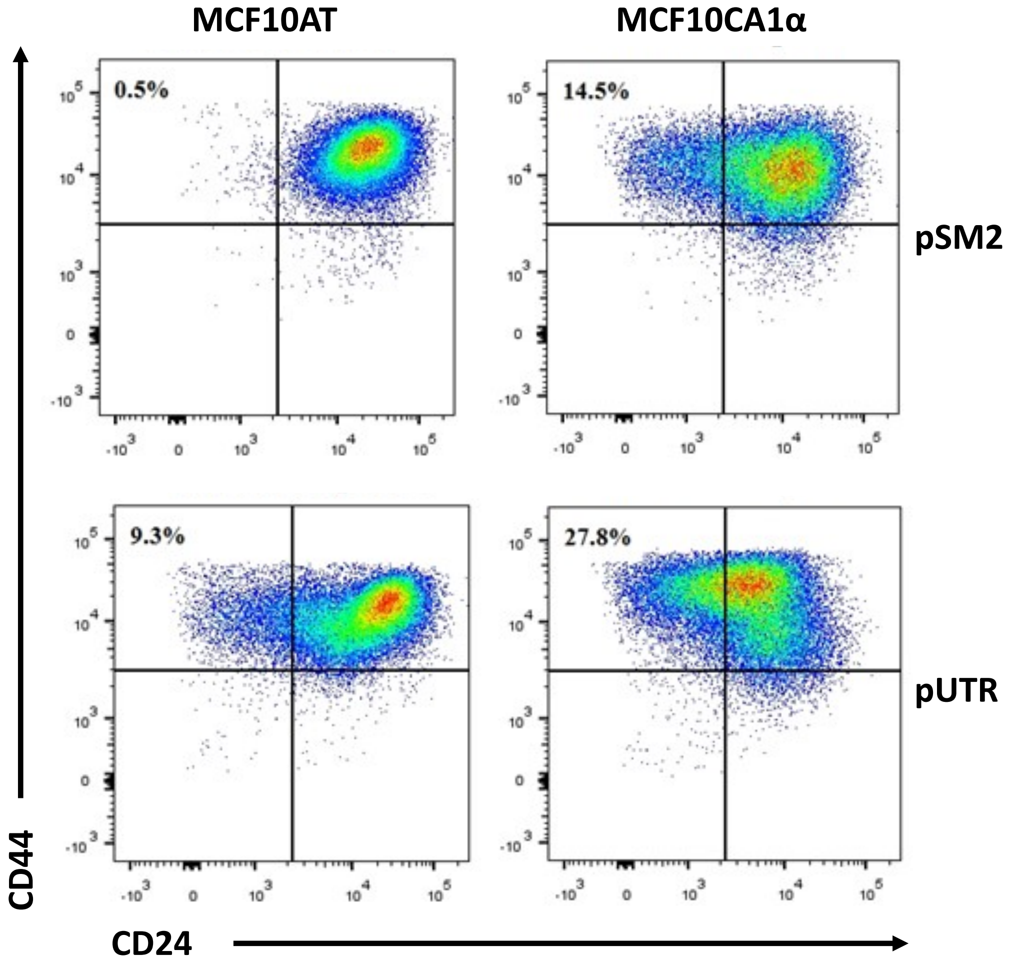

Figure S3: The mesenchymal-like CSCs in MCF10AT-pSM2, MCF10AT-pUTR, MCF10CA1α-pSM2, MCF10CA1α-pUTR cells

A The Sequence of shRNAs in pSM2 vector (Paddison *et al.*, 2004) shRNA insertion site. **B** Flow cytometry analysis for LINE-1 inhibitory cells and their non-functional controls. The dots in the up-left corner of each FACS figure represent the CD44+/CD24- *mesenchymal-like* CSCs. The frame lines were set based on their unstained controls.

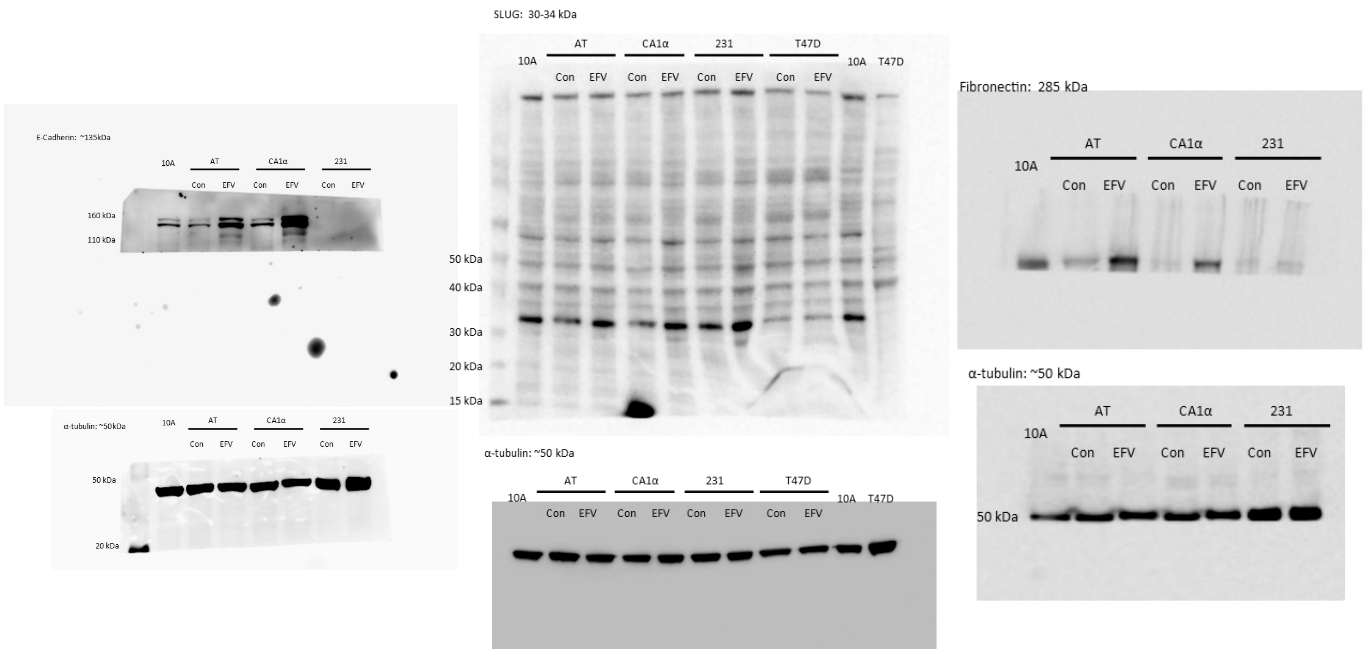

Protein relative fold-change	MCF10AT			MCF10CA1α			MDAMB231		
**E-cadherin**	4.0356	2.6927	2.2197	5.3729	1.3694	5.4450	NA	NA	NA
	p = 0.0677			*p* = 0.1508			NA		
**SLUG**	1.3969	1.8838	1.7449	1.8450	2.2458	1.6661	NA	NA	NA
	p = 0.0430			*p* = 0.0331			NA		
**Fibronectin**	2.5875	2.0114	3.2328	2.5551	3.8212	4.6381	1.1563	2.0730	1.3184
	p = 0.0448			*p* = 0.0478			*p* = 0.2093		

Figure S4: Original E-cadherin, SLUG, and Fibronectin Western blotting results with statistical tests.

Western blotting result images were captured by ImageQuant LAS 4000 biomolecular imager (GE healthcare). The intensity of each band was measured by ImageJ freeware and calculated by Microsoft Excel. Triplicates Western blotting results were collected and *p*-values were calculated by using paired 2-tails Student's t-test as shown in the table.
